# Lipase immobilized on functionalized superparamagnetic few-layer graphene oxide as an efficient nanobiocatalyst for biodiesel production from *Chlorella vulgaris* bio-oil

**DOI:** 10.1186/s13068-020-01688-x

**Published:** 2020-03-20

**Authors:** Tahereh Nematian, Alireza Shakeri, Zeinab Salehi, Ali Akbar Saboury

**Affiliations:** 1 Department of Applied Chemistry, School of Chemistry, College of Science, University of Tehran, Tehran, Iran; 2 Department of Biotechnology Engineering, School of Chemical Engineering, College of Engineering, University of Tehran, Tehran, Iran; 3 Institute of Biochemistry and Biophysics, University of Tehran, Tehran, Iran

**Keywords:** Few-layer graphene oxide, Biodiesel, *Chlorella vulgaris*, Bio-oil, Superparamagnetic, Nano-biocatalyst

## Abstract

**Background:**

Microalgae, due to its well-recognized advantages have gained renewed interest as potentially good feedstock for biodiesel. Production of fatty acid methyl esters (FAMEs) as a type of biodiesel was carried out from *Chlorella vulgaris* bio-oil. Biodiesel was produced in the presence of nano-biocatalysts composed of immobilized lipase on functionalized superparamagnetic few-layer graphene oxide via a transesterification reaction. A hybrid of few-layer graphene oxide and Fe_3_O_4_ (MGO) was prepared and characterized. The MGO was functionalized with 3-aminopropyl triethoxysilane (MGO–AP) as well as with a couple of AP and glutaraldehyde (MGO–AP–GA). The *Rhizopus oryzae* lipase (ROL) was immobilized on MGO and MGO–AP using electrostatic interactions as well as on MGO–AP–GA using covalent bonding. The supports, MGO, MGO–AP, and MGO–AP–GA, as well as nano-biocatalyst, ROL/MGO, ROL/MGO–AP, and ROL/MGO–AP–GA, were characterized using FESEM, VSM, FTIR, and XRD. The few-layer graphene oxide was characterized using AFM and the surface charge of supports was evaluated with the zeta potential technique. The nano-biocatalysts assay was performed with an evaluation of kinetic parameters, loading capacity, relative activity, time-course thermal stability, and storage stability. Biodiesel production was carried out in the presence of nano-biocatalysts and their reusability was evaluated in 5 cycles of transesterification reaction.

**Results:**

The AFM analysis confirmed the few-layer structure of graphene oxide and VSM also confirmed that all supports were superparamagnetic. The maximum loading of ROL (70.2%) was related to MGO–AP–GA. The highest biodiesel conversion of 71.19% achieved in the presence of ROL/MGO–AP–GA. Furthermore, this nano-biocatalyst could maintain 58.77% of its catalytic performance after 5 cycles of the transesterification reaction and was the best catalyst in the case of reusability.

**Conclusions:**

In this study, the synthesized nano-biocatalyst based on bare and functionalized magnetic graphene oxide was applied and optimized in the process of converting microalgae bio-oil to biodiesel for the first time and compared with bare lipase immobilized on magnetic nanoparticles. Results showed that the loading capacity, kinetic parameters, thermal stability, and storage stability improved by the functionalization of MGO. The biocatalysts, which were prepared via covalent bonding immobilization of enzyme generally, showed better characteristics.

## Background

The increasing dependency of the world on petroleum concurrent with the reduction in fossil fuel resources and environmental pollution have persuaded the governments to develop renewable alternatives [1, 2]. The biofuels are of these alternatives which can be used for transportation instead of petroleum-based fuels. They are produced from biosources such as biomass through various processes. The light alcohols and ethers (methanol, ethanol, and diethyl ether), aliphatic and aromatic hydrocarbons, and fatty acid methyl esters can be produced from biomasses [3, 4]. Actually, the carbon content of theses fuels, which is combusted in the engines and released into the atmosphere, has been stabilized from the atmosphere in the masses of the plants. So, the biofuels have zero net carbon release into the environment [5, 6].

Biodiesel is a diesel fuel produced from bioresources. Fatty acid methyl esters (FAMEs) and other mono-alkyl esters of long-chain fatty acids are well-known types of biodiesel. It has been reported that the addition of biodiesel to petroleum diesel can increase the cetane number of fuel and improve the lubrication of engine [7]. The plant oil and animal fat can be converted to this type of fuel through a transesterification reaction. Microalgae because of high oil production efficiency and rapid growth rate and growing ability in the wastewater or non-arable lands has been highly regarded as affordable and economical feedstock for biodiesel production [8]. Among algal species, *Chlorella vulgaris*, due to its lipid-rich biomass content and easy cultivation has great potential as a feedstock for biodiesel production [9–11].

The transesterification reaction for the purpose of biodiesel production is performed using biomass oily content and methanol/ethanol in the presence of catalysts. Catalysts used in this reaction are classified into acidic, basic, and enzymatic [12, 13]. Enzymatic catalysts have been widely used in different industrial applications because of selective performance and easy product separation. Lipases are well-known enzymes used as biocatalysts for the hydrolysis and transesterification of triglycerides [14–16]. The immobilization increases the chemical and thermal stability of the enzyme and can improve its catalytic activity. Furthermore, the enzyme immobilization facilitates catalyst separation at the end of the reaction. The covalent bonding, electrostatic adsorption, encapsulation, and entrapping are common methods for enzyme immobilization [17–19].

Different nanostructure materials have been used as support for the immobilization of enzymes. A variety of nanostructures such as nanoparticles, polymer nanofibers and nanocomposites, metal organic frameworks, nano- and mesoporous silica, carbon nanotubes, graphene, and graphene oxide have been used for enzyme immobilization [20–24]. The magnetic nanoparticles have been known as very useful material in different applications. The Fe_3_O_4_ nanoparticles have been used for pH-responsive drug release system. A core–shell structure has been synthesized using these magnetic nanoparticles and exhibited enhanced chemotherapy efficacy [25]. It has been reported that iron oxide nanoparticles enhance the efficiency of cancer drug delivery systems. They can be leaded to meet targets by means of magnetic field and positively affect drug efficiency [26, 27].

The immobilization of enzymes on the bare and functionalized Fe_3_O_4_ superparamagnetic nanoparticles (MNPs) and MNPs-carbon supports have been investigated. Carboxyl-functionalized graphene oxide was utilized to immobilize lipase. The MNPs sub-microspheres with nano-scale diameters have been functionalized with epoxy chloropropane for the purpose of lipase immobilization. This nano-biocatalyst has been used for biodiesel production from acidified waste cooking oil and the biodiesel production yield of 97.11% has been reported [28]. The amine-functionalized Fe_3_O_4_@C has been synthesized and used for laccase enzyme immobilization. The significant improve has been reported in enzyme loading, operation pH range, and storage stability. The residual activity of 60% obtained after 10 cycles of reuse [29]. The carboxymethyl chitosan functionalized magnetic nanoparticles (Fe_3_O_4_@CM-CTS) have been prepared and used for trypsin immobilization. Trypsin has been successfully immobilized on Fe_3_O_4_@CM-CTS via 1-ethyl-3-(3-dimethylaminopropyl) carbodiimide and glutaraldehyde by covalent bonds. The kinetic studies revealed that the efficient biocatalytic activity trypsin has been retained after immobilization with the maximum catalytic activity of 88.5% [30].

The carbon-based supports have been extensively applied for this aim because of neutral and biodegradable nature, and thermal and chemical stability. The relatively easy functionalization of these structures makes them very suitable for electrostatic adsorption and covalent binding of the enzyme to the support [31–33]. Recently, graphene oxide (GO) has been used in many applications because of its unique properties. The large surface area, high enzyme loading efficiency, thermal stability, having different functional groups, and the modifiability, make GO a very interesting carbon nanomaterial for enzyme immobilization [34, 35].

Recent research has been devoted to the investigation of thermostability and capacity of enzyme loading on graphene oxide. The immobilization of *Brevibacillus borstelensis* lipase on functionalized GO with glutaraldehyde showed a positive and remarkable effect on the thermostability of biocatalyst at 95 ℃ over a broad alkaline pH range of 7–12 [36]. Modified exfoliated graphene oxide via 3-aminopropyl triethoxysilane (AP) has been employed as support for *Candida rugosa* lipase immobilization and used for the synthesis of ester ethyl caprylate. The results showed an improvement in the thermal stability of biocatalyst so that at 40 ℃, enzyme activity has reached 80% for immobilized lipase and 58% for free one [37].

The magnetically recoverable catalysts have been developed for various reactions. The magnetization of catalyst facilitates its separation using an external magnetic field upon the reaction termination. This method has improved the reusability of catalysts and biocatalysts [38, 39]. The immobilization of enzymes on bare and functionalized Fe_3_O_4_ superparamagnetic nanoparticles (MNPs) and MNPs-carbon supports have been investigated. The MNPs-GO hybrid has been used as a sensor, catalyst, and adsorbent for the removal of arsenic, antibiotics, and some other water pollutants [40–43]. The lipase immobilization on magnetic amino-functionalized GO for the aim of biodiesel production from *R. communis* oil has been performed. It has been reported that the immobilization increases the biodiesel up to 78% compared with free lipase [44]. The similar support has been used for the immobilization of *Candida rugosa* lipase for the conversion of soybean oil to biodiesel. The biodiesel production yield of 92.8% and 5 times recyclability of the catalyst has been reported [45].

In this research, superparamagnetic nano-biocatalysts consist of electrostatically and covalently immobilized *Rhizopus oryzae* lipase on MNP and magnetized graphene oxide were synthesized and characterized. The loading capacity, kinetic parameters, thermal and storage stability of these catalysts were evaluated in a model hydrolysis reaction. The biodiesel production from transesterification of *Chlorella vulgaris* microalgae bio-oil was carried out in the presence of these nano-biocatalysts and also the catalyst reusability was investigated.

## Results and discussion

### Characterization

#### Atomic force microscopy (AFM)

**Fig. 1:**
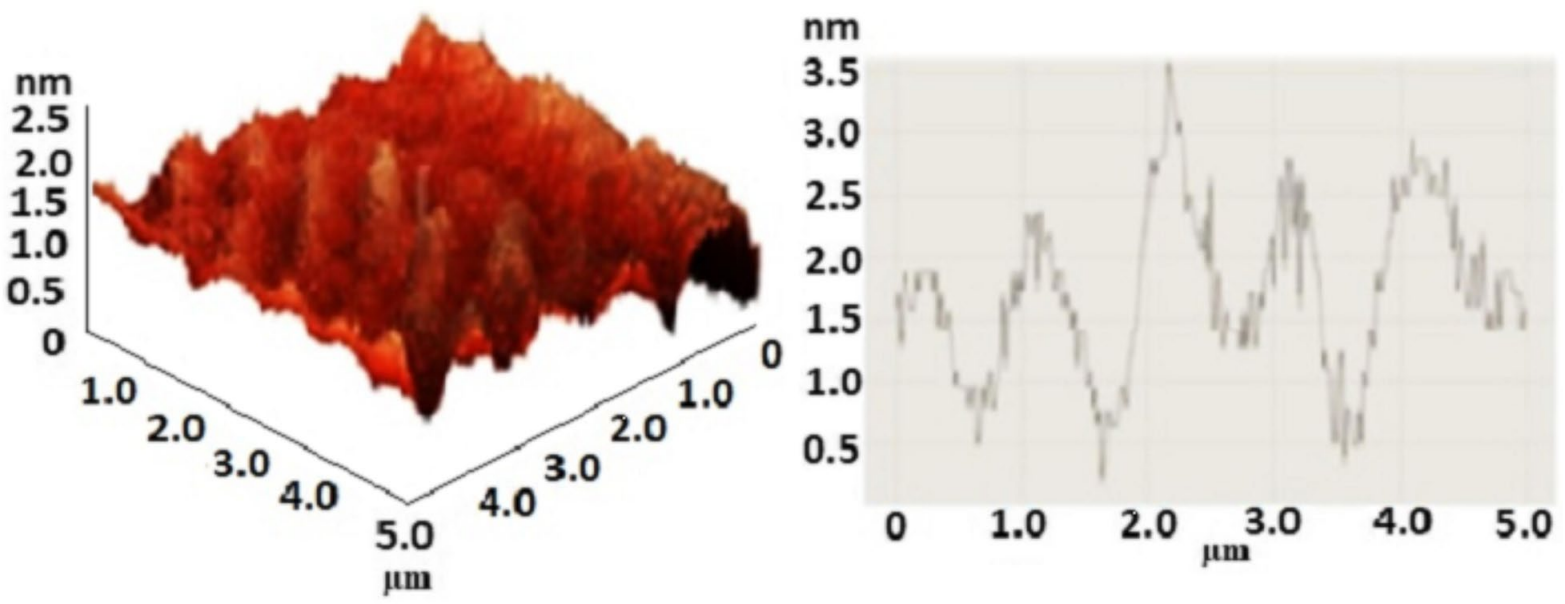
The AFM image of GO sheets and corresponding height profile

Atomic force microscopy (AFM) height profile of the spin-coated sample on the silicon oxide substrate is shown in Fig. 1. The profile revealed a successful exfoliation of graphite layers and demonstrated the few-layer graphene oxide structure. Therefore, the results confirm the synthesis of nanoscale few-layer graphene oxide. As shown in the graph, the thickness of the GO pieces is approximately 2.5 nm. Considering that the thickness of every layer in the graphite structure is 0.8 nm, the achieved GO pieces have almost three layers of carbon sheets [46].

#### FTIR spectra

**Fig. 2:**
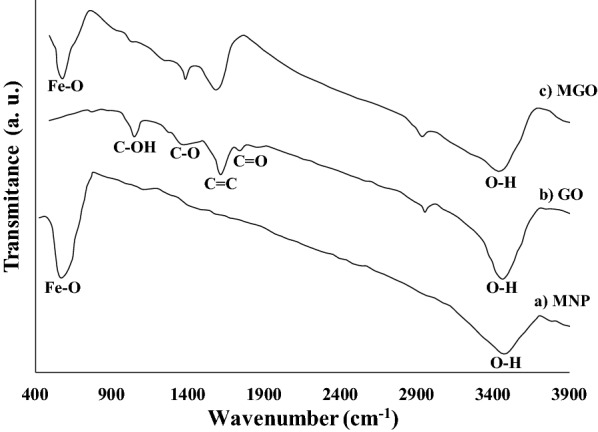
The FTIR spectra of a MNP, b GO, and c MGO

**Fig. 3:**
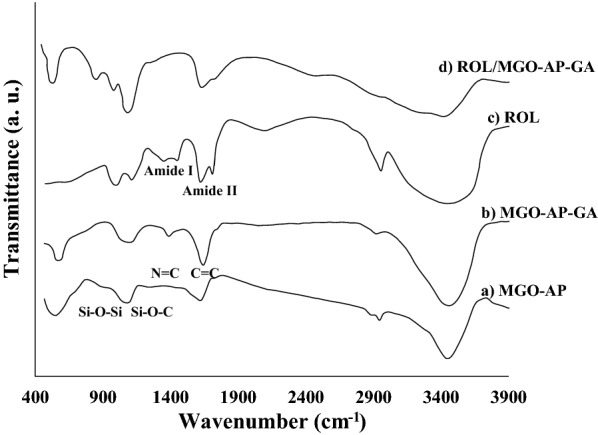
The FTIR spectra a MGO–AP, b MGO–AP–GA, c ROL, and d ROL/MGO–AP–GA

**Fig. 4:**
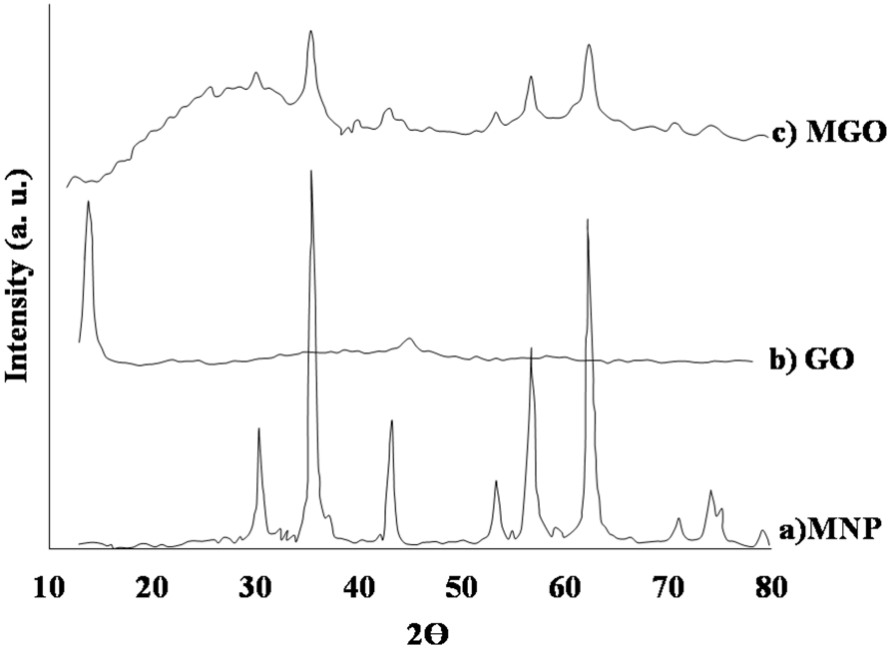
The XRD patterns of a MNP, b GO, and c MGO

Figure 2 shows the FTIR spectra of a) MNP, b) GO, and c) MGO. For the bare MNP (Fig. 2a), two dominant peaks were appeared at 567 cm^−1^ and 3400 cm^−1^ which are related to Fe–O and O–H bonds, respectively. In the GO spectrum (Fig. 2b), the broad peak around 3400 cm^−1^ is attributed to O–H stretching vibration indicating the presence of O–H and COOH functional groups in the GO structure. The presence of oxygen-containing functional groups, such as C = O stretching vibration of carboxylic and carbonyl groups, C–O stretching vibration of an epoxy group and C–OH group was demonstrated with the peaks appeared at 1732, 1252 and 1040 cm^−1^, respectively. Furthermore, the presence of C = C bonds in aromatic rings on GO plates was demonstrated with the peak appeared at 1611 cm^−1^ [47]. The spectrum of MGO showed a peak that appeared at 574 cm^−1^. It is related to the Fe–O bond and can be detected in all the magnetic nanostructures spectrums (Figs. 3, 4).

Figure 3 shows the FTIR spectra of a) MGO–AP, b) MGO–AP–GA, c) ROL, and d) ROL/MGO–AP–GA. In the first functionalization step of MGO (Fig. 3a), the appearance of peaks at 1094 and 1034 cm^−1^ are related to Si–O–C and Si–O–Si bonds and confirmed the functionalization of MGO with amine groups. The bonds in the range of 2850–2920 cm^−1^ ascribed to CH_2_, provide another evidence of AP attachment [48]. In the MGO–AP–GA spectrum (Fig. 3b), enhancement of absorption peaks at 1635 cm^−1^ can be related to the N = C covalent bond formation between glutaraldehyde and AP. Also, the peak at 1740 cm^−1^ can be related to C = O aldehyde groups of glutaraldehyde. The FTIR spectrum of ROL (Fig. 3d) indicated two characteristic peaks in the wavenumbers of 1610 cm^−1^ and 1454 cm^−1^. The peak at 1610 cm^−1^ associated with the C = O stretching vibration in amide-type I and 1454 cm^−1^ associated with the N–H bending and C–N stretching vibrations in amide-type II [49]. The related peaks of amide-type I and amide-type II are attributed to the protein backbone of the enzyme. Broadening and increasing intensity of the characteristic absorption peaks of ROL in the FTIR spectrum of ROL/MGO–AP–GA (Fig. 3d) confirmed successful immobilization of ROL on MGO–AP–GA [50].

#### XRD patterns

**Fig. 5:**
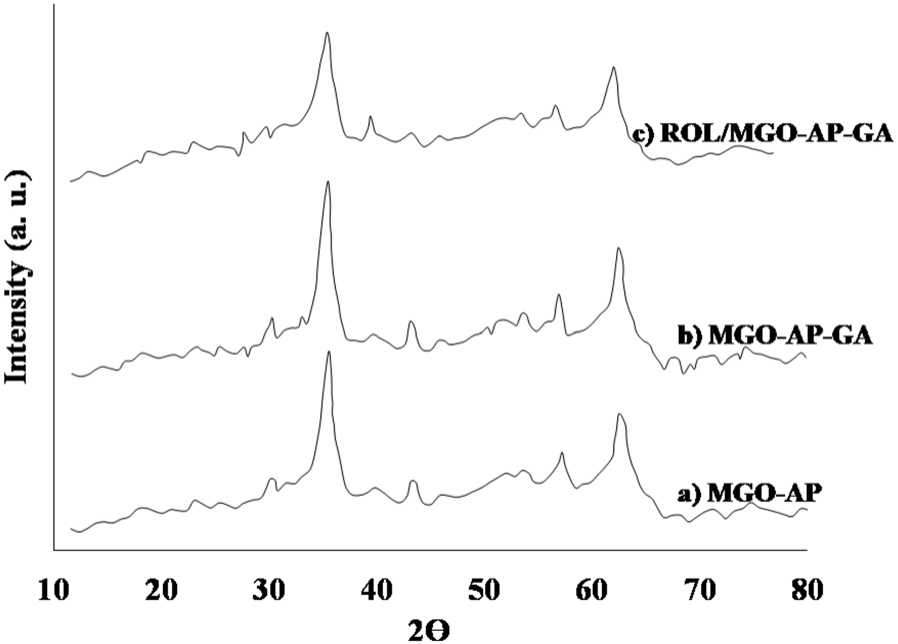
The XRD patterns of a MGO–AP, b MGO–AP–GA, and c ROL/MGO–AP–GA

**Fig. 6:**
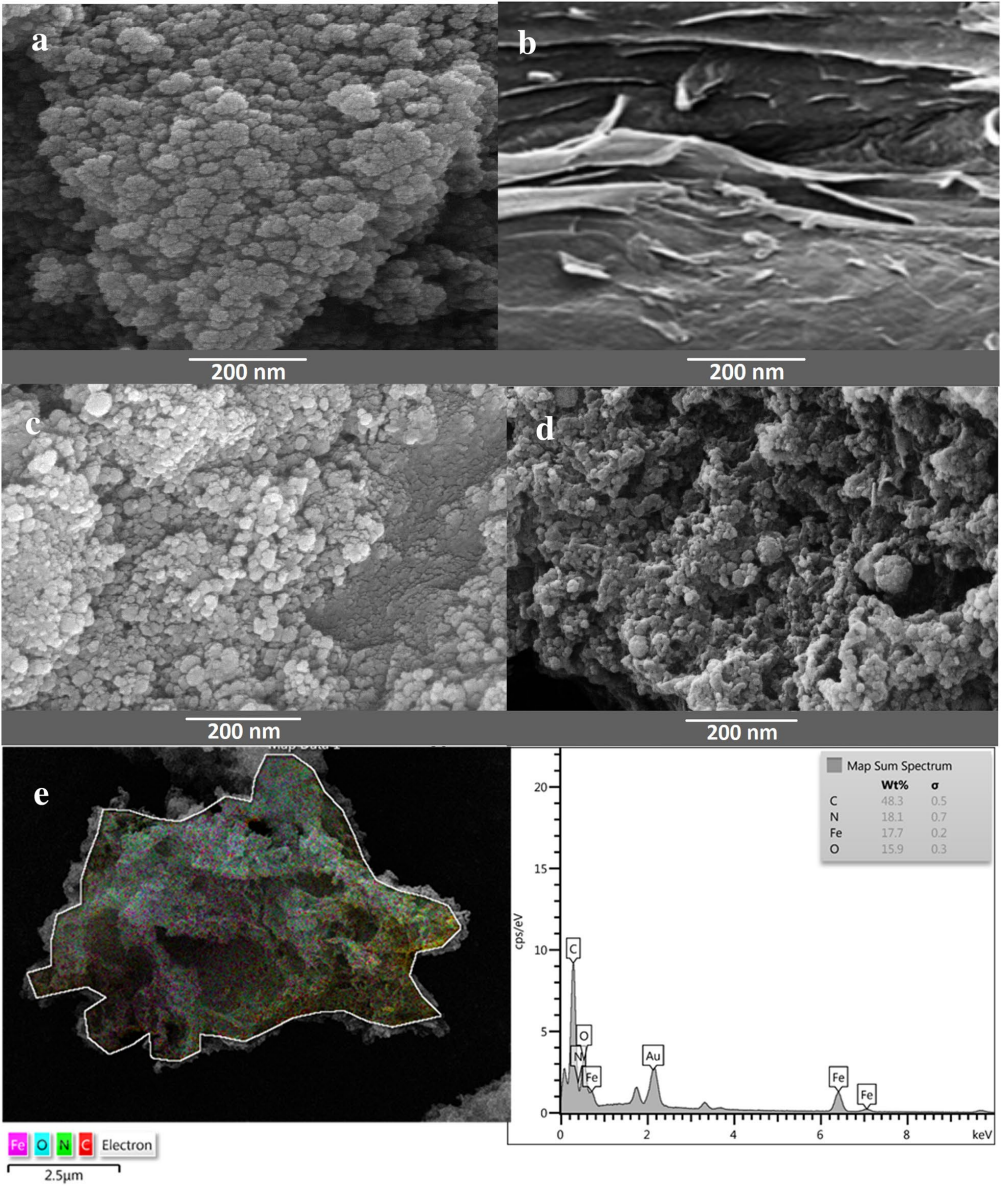
The FESEM micrographs of a MNP, b GO, c MGO, and d ROL/MGO–AP–GA as well as the EDS elemental mapping of e ROL/MGO–AP–GA

Typical XRD patterns for synthesized supports are presented in Figs. 5, 6. The existence of six characteristic peaks at 2*θ* of 30.0°, 35.4°, 43.1°, 53.5°, 57.0°, and 62.6° in the spectrum of MNP (Fig. 4a) indicated the formation of the crystalline structure of MNP. The XRD pattern of GO in Fig. 4b shows a strong and sharp peak at 2*θ* = 10.74°, which is related to the exfoliated GO with a d-spacing of 0.84 nm [51]. All diffraction peaks in the XRD pattern of MGO (Fig. 4c) are related to the MNP crystalline phase. The characteristic peak of GO was broadened and weakened due to the exfoliation of GO during the MGO synthesis process. As shown in Fig. 5, the appeared peaks in XRD patterns of MGO–AP, MGO–AP–GA, and ROL/MGO–AP–GA are similar to the MGO peaks which means that functionalization and immobilization process has no effect on the crystallinity of MGO.

#### FESEM micrographs

**Fig. 7:**
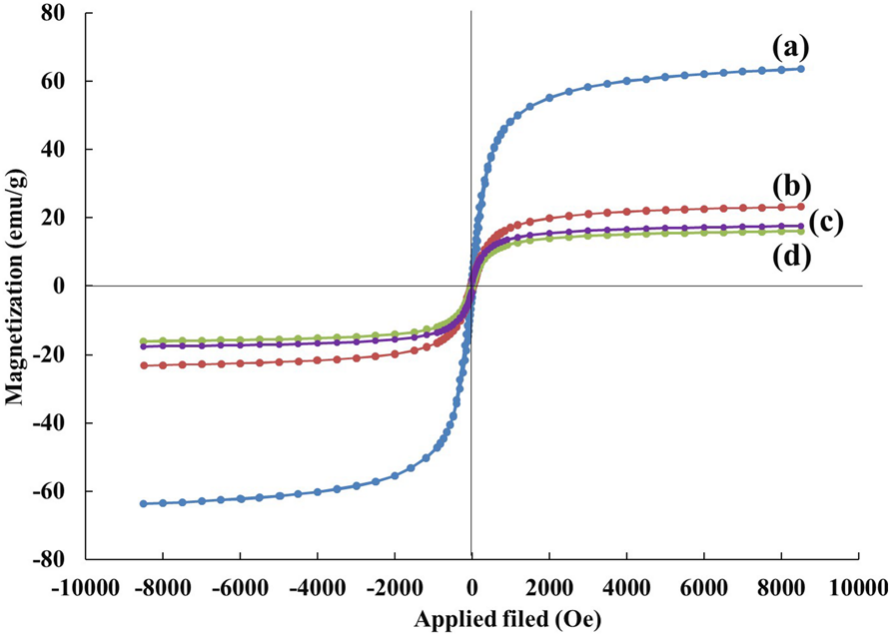
The VSM analysis for a MNP, b MGO, c MGO–AP and d MGO–AP–GA

The FESEM was employed to characterize the morphology of the prepared MNP, GO and MGO. Also, the FESEM and EDS elemental mapping were used for evaluation of morphology and elemental distribution on ROL/MGO–AP–GA, as the nano-bio catalyst with the best performance (Fig. 6). As shown in Fig. 7a, the spherical shape of MNP is obvious and the particle size is in range of 20–30 nm. The smooth and sheet-like structure of GO can be observed in Fig. 6b. Figure 6c shows the FESEM micrograph of MGO. The created wrinkles and the spherical-shape MNP nanoparticles are clearly obvious on the surface of GO. Furthermore, the size range of MNP on the GO sheets is relatively as same as the bare MNP. Figure 6d illustrates the FESEM micrograph of ROL/MGO–AP–GA. The brighter fine zones that are visible on the surface of MGO in FESEM micrograph can be related to ROL immobilization on MGO–AP–GA. The EDS elemental mapping of ROL/MGO–AP–GA shows a good distribution of elements on the nano-bio catalyst (Fig. 6c).

#### Vibrating sample magnetometer (VSM)

Vibrating sample magnetometer (VSM) analysis was used for the evaluation of magnetic properties of synthesized supports. As shown in Fig. 7, the magnetic hysteresis loop for all curves was S-like shape over the applied magnetic field at room temperature, indicating that the samples were superparamagnetic. The magnetic response of MNP, MGO, MGO–AP, and MGO–AP–GA were 63.67, 23.19, 17.56 and 16.06 emu g^−1^, respectively. Depletion in saturation magnetization (*M*_*s*_) of MGO compare with the bare MNP can be attributed to the relatively low MNP mass ratio in the MGO hybrid. The hybrid magnetization changes with the different MNP to GO mass ratios. After AP and GA grafting on MGO, the *M*_*s*_ value was decreased due to the introduction of non-magnetic components to the magnetic one.

#### Zeta potential measurement

The zeta potential measurement was carried out in phosphate buffer medium (100 mM, pH 7.5) to evaluate the surface charge of synthesized supports. As shown in Table 1, the zeta potential of MGO was − 33.58 which more negative than of MNP. The more negative charge of MGO than MNP is related to hydroxyl and carboxyl groups on MGO surface. After the modification of MGO with AP, a slight increase of zeta potential was observed. It can be related to this fact that amine groups contain less negative charge than hydroxyl and carboxyl groups [52]. The zeta potential of the MGO–AP–GA decreased to − 20.24 when the GA was added to the MGO–AP.

**Table 1:** Zeta potential measurement of immobilized ROL

Samples	Zeta potential (mV)
MNP	− 25.84
MGO	− 33.58
MGO–AP	− 17.46
MGO–AP–GA	− 20.24

#### BET surface area

The value of surface area of MNPs and MGO were measured using single point BET. The specific surface area for them were 84 and 288 m^2^ g^−1^ for MNPs and MGO, respectively. The higher surface area of MGO is related to the hybridizing Fe_3_O_4_ magnetic nanoparticles and graphene oxide.

### Nano-biocatalysts assay

#### Loading capacity

**Fig. 8:**
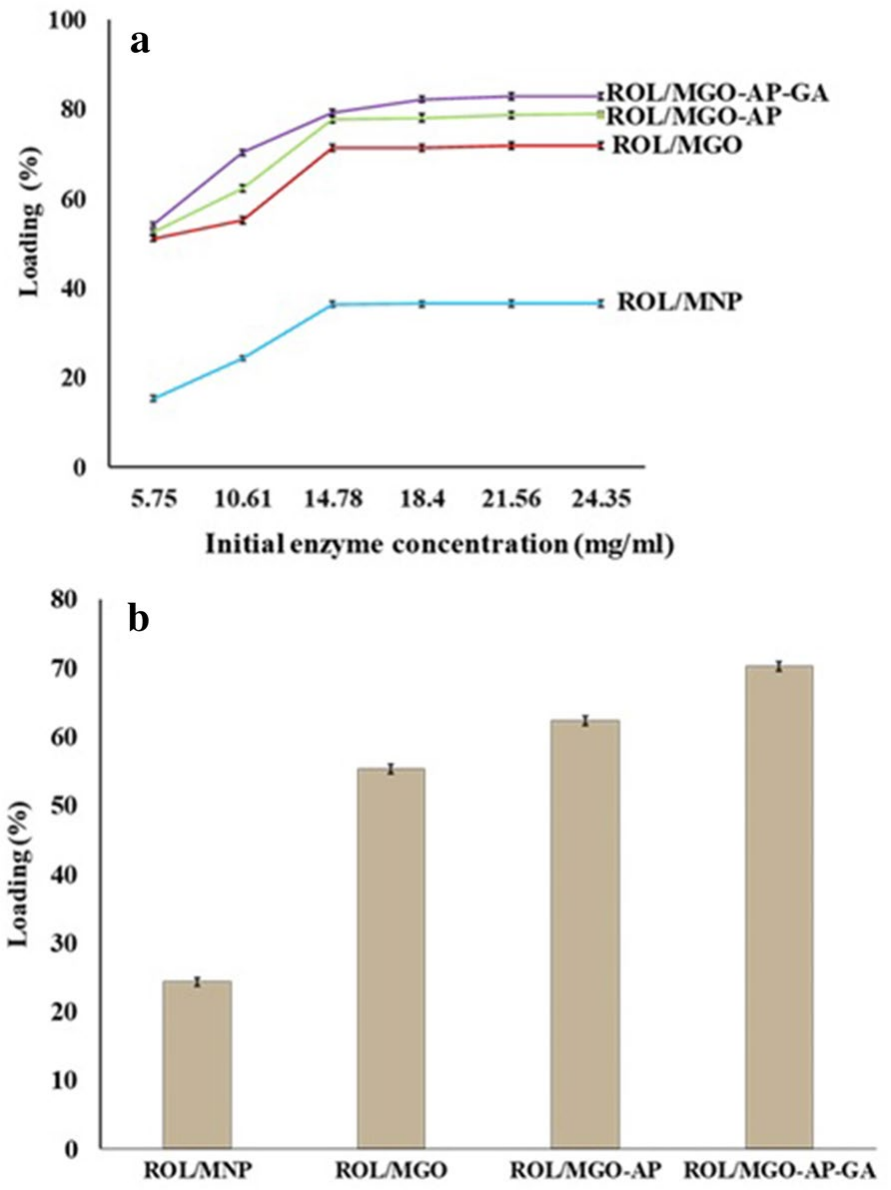
Loading capacity of supports for different ROL initial concentrations **a** and the comparison of supports maximum capacity **b**

The loading capacity of immobilized ROL on the supports was evaluated using Bradford’s method and results are reported in Fig. 8. For this purpose, the supports were exposed to enzyme solutions with different ROL initial concentrations. As shown in Fig. 8a, loading capacity increased with increasing of initial ROL concentration and then reached a maximum value for each support. However, the enzyme loading showed no significant increase for the solutions with concentrations upper than 21 mg mL^−1^ and 14 mg mL^−1^ for MGO–based supports and bare MNP, respectively. Saturation of supports because of their limited capacity is the reason of this observation [53]. The optimum loading percentage of ROL on the supports was varied from 24.23 ± 0.55 to 70.20 ± 0.69 wt% (Fig. 8b). The MGO–AP–GA showed the highest ROL loading capacity and the lowest was attributed to MNP. It can be related to the lower surface area of MNP compared with MGO–based supports. Also, the functionalization of MGO with AP and GA increased loading capacity due to creating a wider spherical area for enzyme attachment.

#### Relative activity

**Fig. 9:**
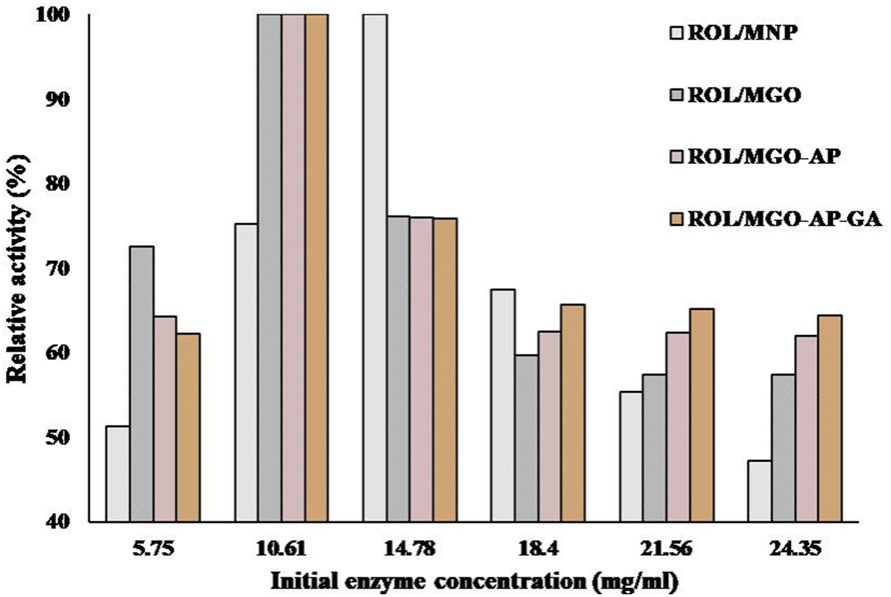
The effect of ROL initial concentration on the relative activity of nano-biocatalysts

Figure 9 shows the effect of the ROL initial concentration on the relative activity of nano-biocatalysts. The prepared nano-biocatalysts in the process of loading capacity tests were used for relative activity evaluation. The relative activity was defined as the ratio of each sample activity to its maximum activity [54]. The increasing of initial ROL concentration caused relative activity enhancement up to the maximum value (100%). The more loaded enzyme and subsequently, more enzyme–substrate contacts in the reaction increased the relative activity. In the case of MNP, when the ROL initial concentration was 14.78 mg mL^−1^, the loading capacity and relative activity were maximum. For MGO–based nano-biocatalysts, the highest relative activity was obtained for ROL initial concentration of 10.61 mg mL^−1^ and was not equal to the maximum loading capacity. More increasing in ROL initial concentration causes the accumulation of enzyme molecules on supports surface. Therefore, the substrate accessibility to enzymes is limited and the relative activity decreases [53]. As a comparison among different supports, by increasing initial enzyme concentration, especially from 14.78 to 24.35 mg mL^−1^, the addition of AP and GA to MGO could increase the relative activity of nano-biocatalyst. It can be related to the fact that the attachment of these two molecules to MGO provides a wider space for enzyme immobilization. Although the enzyme accumulation occurs for each of MNP, MGO, MGO–AP, and MGO–AP–GA support during the loading process and the relative activity decreases by increasing initial enzyme concentration, but lower accumulation occurs for the support with longer molecular chain and wider space for enzyme attachment.

#### Kinetic parameter

The Michaelis–Menten kinetic parameters for the hydrolysis of *p*-PNP in the presence of free and immobilized ROL were measured and presented in Table 2. The *K*_*m*_ is the substrate concentration in which the reaction rate is at half of its maximum velocity and shows an affinity of attachment between enzyme and substrate. The ROL immobilization on the supports caused an obvious reduction in *K*_*m*_ value which indicating more enzyme–substrate affinity of attachment. The *K*_*m*_ was decreased by more than fourfold after attachment of AP and GA to MGO. The less negative *ζ* potential of MGO–AP than MGO which is due to the existence of amine groups in AP structure, as well as the increasing of surface hydrophilicity due to the GA attachment can be responsible.

**Table 2:** The kinetic parameters of free and immobilized ROL

	*K*_*m*_ (mM)	*V*_max_ (µM min^−1^)	*k*_cat_ (min^−1^)	*k*_cat_/*K*_*m*_ (min^−1^ mM^−1^)
ROL	16.45 ± 0.46	541.52 ± 26.68	115.22 ± 7.94	7.01
ROL/MNP	12.25 ± 0.54	212.77 ± 11.11	45.26 ± 4.26	3.69
ROL/MGO	12.67 ± 0.62	336.98 ± 12.42	71.69 ± 6.35	5.91
ROL/MGO–AP	4.01 ± 0.22	357.23 ± 8.24	76.55 ± 5.18	18.95
ROL/MGO–AP–GA	3.04 ± 0.18	369.12 ± 9.83	78.53 ± 6.76	25.83

The maximal velocity (*V*_max_) is defined as the maximum rate of reaction when the enzyme active sites are saturated with substrate and the *k*_cat_ is calculated by dividing the *V*_max_ to the ROL concentration in the reaction mixture. As shown in Table 2, *V*_max_ and *k*_cat_ decreased after ROL immobilization on the supports due to the substrate diffusion limit. This behavior has been observed for enzymes which immobilized on different carriers [55–57]. However, because of the larger surface area of MGO, the mass transfer limit is reduced when MGO is used as support and *V*_max_ and *k*_cat_ are increased compared with MNP. According to the *k*_cat_ to *K*_*m*_ ratio which is an estimation of catalytic efficiency, a significant increase was observed in ROL immobilized on functionalized MGO supports indicating higher catalytic efficiency. The *k*_cat_/*K*_*m*_ value for ROL/MGO–AP–GA was 25.83 which was about fourfold more than ROL/MGO. It indicates that covalent bonding between the ROL and MGO–AP–GA can be considered as an efficient method for lipase immobilization.

#### Thermal stability

**Fig. 10:**
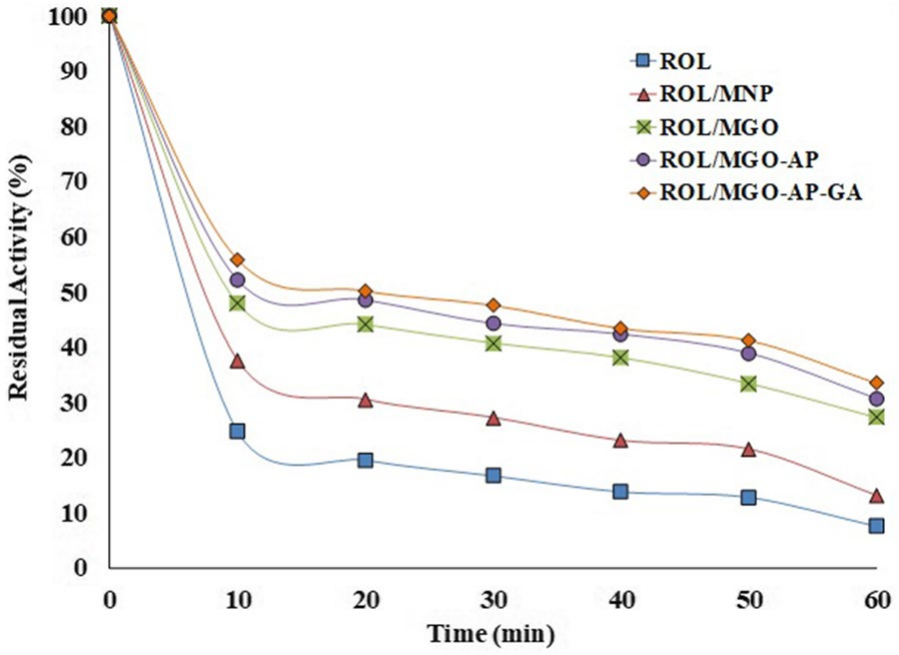
The time-course thermal stability of free and immobilized ROL

The time-course thermal stabilities of all nano-biocatalysts were studied by incubating them at 60 ℃ in different time durations (10 to 60 min) and evaluation of their residual activities (Fig. 10). The residual activity was measured at the optimum conditions after cooling in ice for 30 min and mentioned as thermal stability. Nano-biocatalysts residual activity which incubated at 60 ℃ for 0 min considered as control (with 100% activity). As shown in Fig. 10, the residual activity of free ROL was decreased by increasing the incubation time. According to the figure, the thermal stability of ROL/MGO is more than ROL/MNP which is due to the more functional groups on MGO compare with MNP. After the functionalization of MGO with AP, electrostatic attractions between ROL and support increased and led to a noticeable improvement in the thermal stability of nano-bio catalyst. The residual activities of ROL/MNP, ROL/MGO, ROL/MGO–AP, and ROL/MGO–AP–GA after 60 min incubation and at 60 ℃ were 13.09%, 27.20%, 30.59%, and 33.44%, respectively. The ROL/MGO–AP–GA has the highest thermal stability and it demonstrated that enzyme more attraction with support causes more thermal stability. It can be related to the lower flexibility of the enzyme structure when it has more attractions with the support. The lower flexibility makes the enzyme more resistant to the deformation if the temperature keeps on increasing [58].

#### Storage stability

**Fig. 11:**
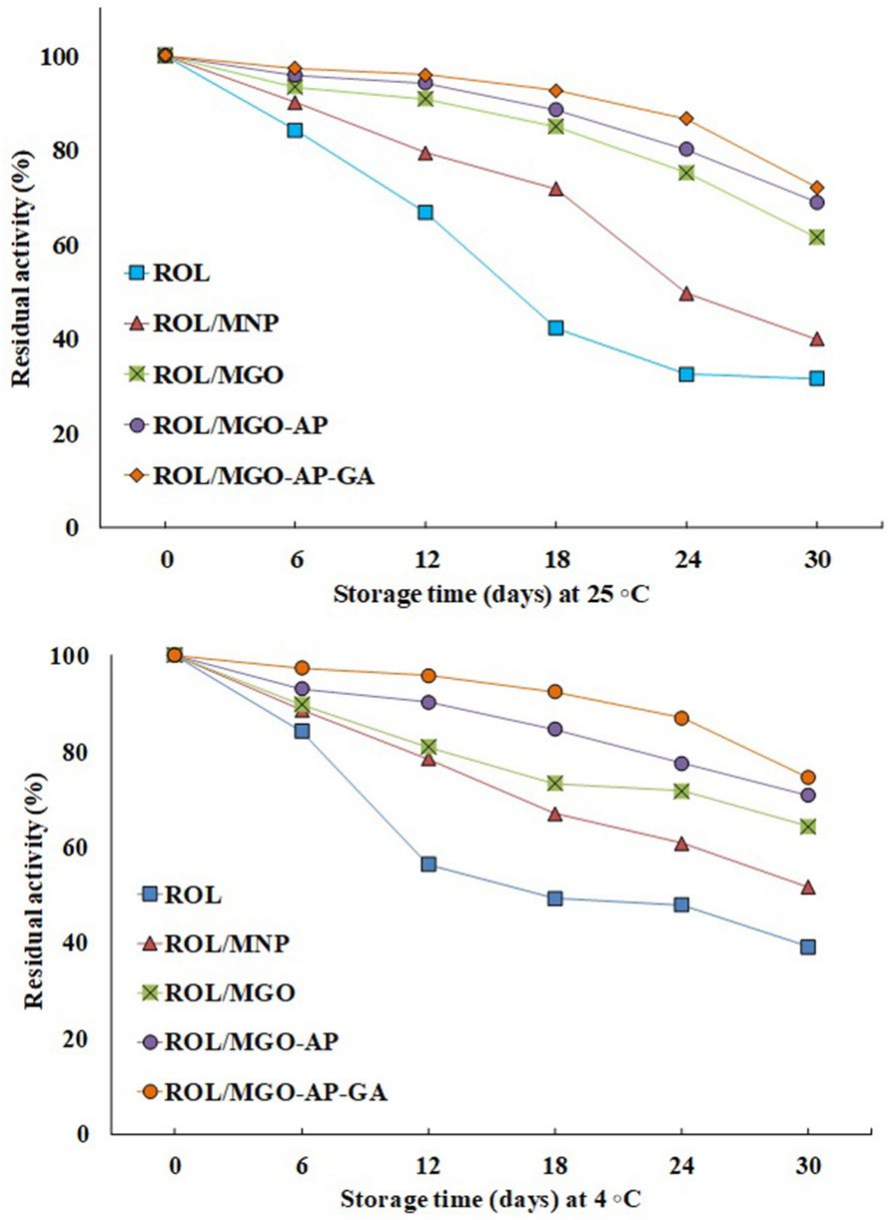
The storage stability of free and immobilized ROL at 25 ℃ and 4 ℃

To evaluate the immobilization efficiency, the storage stability of the immobilized enzyme should be considered as an important requirement. The storage stabilities of the free and immobilized ROL were evaluated at two different incubation temperatures (4 ℃ and 25 ℃). The results are presented in Fig. 11. As shown, for the free ROL at 4 ℃, almost 39.13% of initial activity remained after 30 days, while the activities reached about 51.63%, 64.27%, 70.72% and 74.49% of initial activity for ROL/MNP, ROL/MGO, ROL/MGO–AP, and ROL/MGO–AP–GA, respectively. Maximum storage stability was related to ROL/MGO–AP–GA which can be attributed to ROL covalent bonding to support. This covalent bonding prevents the conformational change of enzyme and consequently helps to preserve its catalytic activity [59]. Figure 11 also shows the storage stability of nano-biocatalysts at room temperature. The behavior was similar to the examination at 4 ℃. However, a total decreasing of storage stability occurred at 25 ℃ compare with 4 ℃.

#### Biodiesel production

**Fig. 12:**
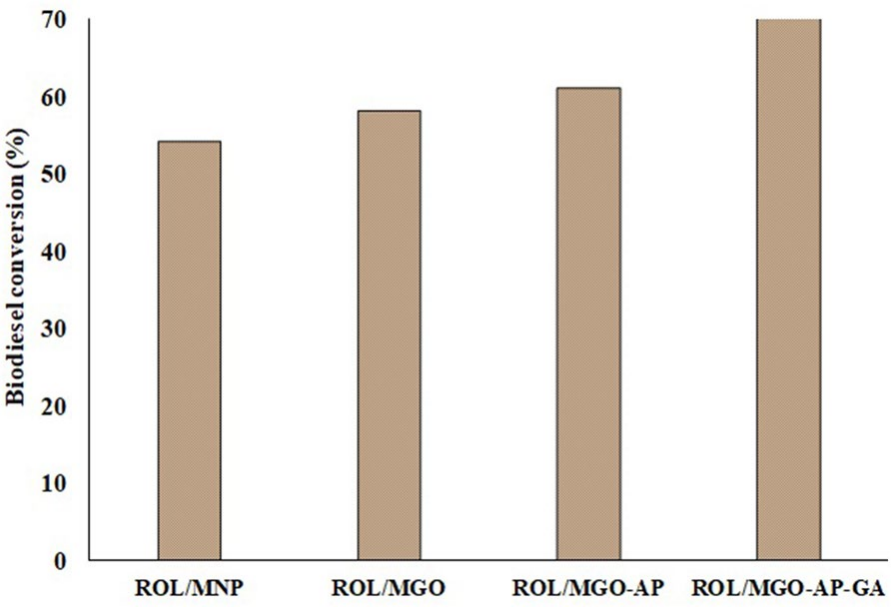
The biodiesel conversion in the presence of free and immobilized ROL

GC–MS technique was used for products to analyze. Four FAMEs (Methyl palmitate, Methyl stearate, Methyl (7*E*,10*E*)-7,10-hexadecadienoate and Methyl (9*Z*,12*Z*,15*Z*)-9,12,15-octadecatrienoate) as biodiesel were detected in the product mixture via GC–MS. The results of *Chlorella vulgaris* oil conversion to biodiesel in the presence of immobilized ROL are represented in Fig. 12. The reaction in the presence of ROL/MNP and ROL/MGO showed the lowest biodiesel conversion of 54.14% and 57.05%, respectively. Since ROL is attached to MNP and MGO via comparatively weak physical adsorption, it is prone to enzyme leaching. Leaching of enzyme may provide a homogenous reaction medium and perform higher reactivity. However, the presence of n-hexane and the impurities in the extracted microalgae oil, can strongly deactivate *Rhizopus oryzae* lipase in the reaction medium after leaching. Actually, the immobilization of enzyme using covalent bonding prevents its denaturation during reaction. So, leaching and consequently denaturation (deactivation) of enzyme can be the reason of lower conversions in the presence of physically immobilized ROLs. Hence it can result in low FAMEs production in comparison with two other nano-biocatalysts. However, a slight general increase of ROL/MGO biodiesel conversion was occurred in order to higher catalytic efficiency of ROL/MGO compared with ROL/MNP.

Biodiesel conversion increased in the presence of ROL/MGO–AP. Amine groups of AP improve the electrostatic interactions due to the negative charge decreasing of the MGO surface as well as decreases the steric hindrance of the enzyme. Therefore, enzyme leaching is less likely to occur and subsequently higher productivity. The highest FAMEs conversion was attributed to ROL/MGO–AP–GA. The more stable and resistant biocatalyst in the harsh reaction conditions was prepared by the functionalization of MGO by AP and GA. The aldehyde groups in GA react with amine groups of ROL to create covalent double bond C = N. The bonds prevent changing ROL conformation during reaction time. In addition, the introducing of AP and GA to the supports facilitates the formation of an active complex between enzyme active sites and substrate via creating a larger polar area around the support surface.

#### Nano-bio catalysts reusability

**Fig. 13:**
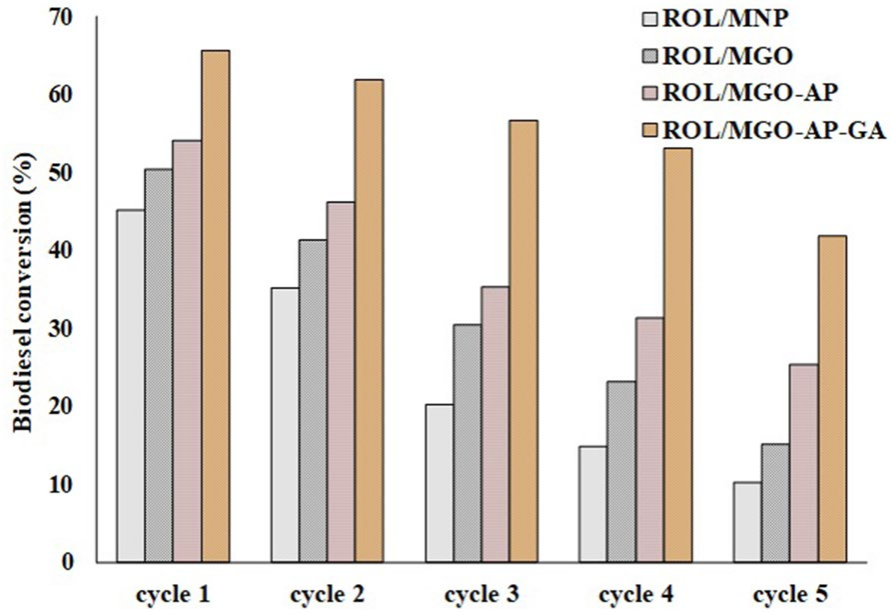
The reusability of ROL/MNP, ROL/MGO, ROL/MGO–AP, and ROL/MGO–AP–GA

The reusability of nano-biocatalysts was evaluated and results are represented in Fig. 13. The best reusing performance was obtained for ROL/MGO–AP–GA among other nano-biocatalysts. The 58.77% of FAMEs conversion was maintained after five cycles of reactions in the presence of ROL/MGO–AP–GA. The results have proved that covalent bonding between the ROL and support helps the physical strength of nano-bio catalyst to be better. So, the nano-bio catalyst stability was increased and consequently higher biodiesel conversion was obtained over multi-cycle reuse.

In a recent research, A cross linked lipase has been synthesized and immobilized on the magnetic amino-functionalized graphene oxide nanocomposites. The reported results for its temperature range of activity was about 40–60 ℃ and for its recyclability after 5 cycles was 70% remained activity. The storage stability has been reported 75% after 30 days of incubation [44]. In another work, the magnetic graphene oxide has been used for immobilization of *Candida rugosa* lipase for biodiesel production. It has been reported that this nano-biocatalyst suffered 50% reduction of relative activity at 80 ℃ while free lipase had no activity in this temperature. The reusability experiments result of this immobilized lipase showed that after 5 cycles the production yield decreased from 92.8 to 75.8% [60].

## Conclusion

Superparamagnetic nano-biocatalysts composed of immobilized *Rhizopus oryzae* lipase on graphene oxide supports were used for producing FAMEs from *Chlorella vulgaris* microalgae oil. This microalga was cultivated in the modified BG-11 medium to increase total biomass lipid (35 wt%). Electrostatic attractions and covalent bonds of ROL to MGO were created via AP and AP–GA as coupling agents. Kinetics parameter, loading capacity, thermal and storage stability of MGO–based nano-biocatalysts were evaluated and compared with free ROL and ROL/MNP. Hybrids of GO and magnetite nanoparticles which used as lipase immobilization supports could increase the catalytic performance of enzyme compare with the bare magnetite nanoparticles. The modification of MGO with AP and AP–GA could enhance the enzyme loading and catalytic efficiency. It can be related to creating an appropriate distance between the support and enzyme. The wider space can reduce steric hindrance as well as fully prevent enzyme intermolecular crosslinking. The catalytic efficiency of ROL/MGO–AP–GA was 4 times higher than the free ROL. The covalent bonding in ROL/MGO–AP–GA caused the biocatalyst more stable and resistant in the microalgae oil transesterification reaction medium. Therefore, the highest biodiesel conversion and the best reusability performance were presented by ROL/MGO–AP–GA.

## Methods

### Materials

For the preparation of graphene oxide (GO) according to the modified Hummer’s method, the chemicals including, sulfuric acid (> 98%), ortho- Phosphoric acid (85%), potassium permanganate (KMnO_4_), hydrogen peroxide (30%), and hydrochloric acid (37%), were purchased from Merck. Pristine graphite powder was supplied from Fluka Company. The precursors for the synthesis of the functionalized MGO were iron (II) sulfate tetrahydrate (FeSO_4_·4H_2_O, 99.7%), iron (III) chloride hexahydrate (FeCl_3_·6H_2_O, 99.0%), ammonia (NH_4_OH, 25 wt%), absolute ethanol, 3-aminopropyl triethoxysilane (AP), and glutaraldehyde (GA) and were purchased from Merck. *Rhizopus oryzae* lipase (light brown powder, ≥ 30,000 U/g) was purchased from Sigma–Aldrich. Reagents for the nano-biocatalysts assay, *p*-nitrophenyl palmitate (*p*-NPP), isopropyl alcohol (99.7%), bovine serum albumin (BSA), Coomassie Brilliant Blue G-250, Triton X-100, and Arabic gum were purchased from Merck. BG-11 medium (Blue-Green medium) components, for the cultivation and maintenance of the microalgae *Chlorella vulgaris CCAP* were obtained from either Merck. All organic solvents and other chemicals purchased from Merck and were of analytical grade.

#### Magnetic nanoparticles preparation

MNPs were synthesized using co-precipitation of FeCl_3_·6H_2_O and FeSO_4_·4H_2_O with a 2:1 molar ratio in 90 cm^3^ deionized water. The aqueous solution was stirred continuously under a nitrogen atmosphere at 85 ℃. To achieve a solution with pH = 9, a certain amount of 25% ammonia solution was added to it dropwise. After 1 h, the obtained black sediment (MNP) was collected using an external magnetic field and washed several times with deionized water.

#### Graphene oxide and magnetic graphene oxide preparation

Graphene oxide (GO) was synthesized using graphite flakes through the modified Hummers’ method. One gram of graphite flakes was dispersed in a 9:1 mixture of H_2_SO_4_/H_3_PO_4_ and stirred for 30 min. The reaction medium was heated up to 50 ℃. Then 6 g of KMnO_4_ was added slowly to this mixture and stirred 24 h at 50 ℃. The mixture was cooled and maintained below 20 ℃, afterward. Subsequently, 200 mL of frozen deionized water was added gradually to it. The suspension was further treated with hydrogen peroxide 30% (5 mL). The solution color turned into yellow and it determined that the reaction was completed. The suspension was washed with HCl (1 M) to remove any residual ions and acids from the reaction medium. The obtained GO was washed using a 5:1 V/V water:ethanol several times by applying centrifugal force.

Magnetic GO (MGO) was prepared via an in situ deposition of Fe_3_O_4_ nanoparticles on the surface of GO with a 1:1 initial mass ratio of GO and Fe_3_O_4_. In a three-necked flask with a flow of N_2_, 0.05 g of FeCl_3_·6H_2_O and 0.1 g of FeSO_4_·4H_2_O were dissolved in 50 mL deionized water. The reaction was followed by the addition of 10 mL GO (1 mg mL^−1^) to the solution and heated to 80 ℃. After 15 min, 10 mL of 25% ammonia solution was added to the solution until the pH reached around 9. The reaction was carried out at 85 ℃ for 1 h. The black sediment was collected using an external magnetic field and then washed with deionized water three times until the pH was neutralized.

#### Functional magnetic graphene oxide preparation

For functionalization of MGO with 3–aminopropyl triethoxysilane (AP), 0.05 g MGO was dispersed in 4.6 mL of ethanol and sonicated under 70 kHz frequency. The 0.1 mL of acetic acid and 0.1 mL of AP were added to this mixture. After 5 h shaking at room temperature, MGO–AP was prepared. The product was separated using an external magnetic field and washed with deionized water. To functionalize MGO with both AP and GA (MGO–AP–GA), 0.04 mL of GA solution was added to the synthesized MGO–AP and shaken for 2 h at room temperature.

#### Enzyme immobilization

**Fig. 14:**
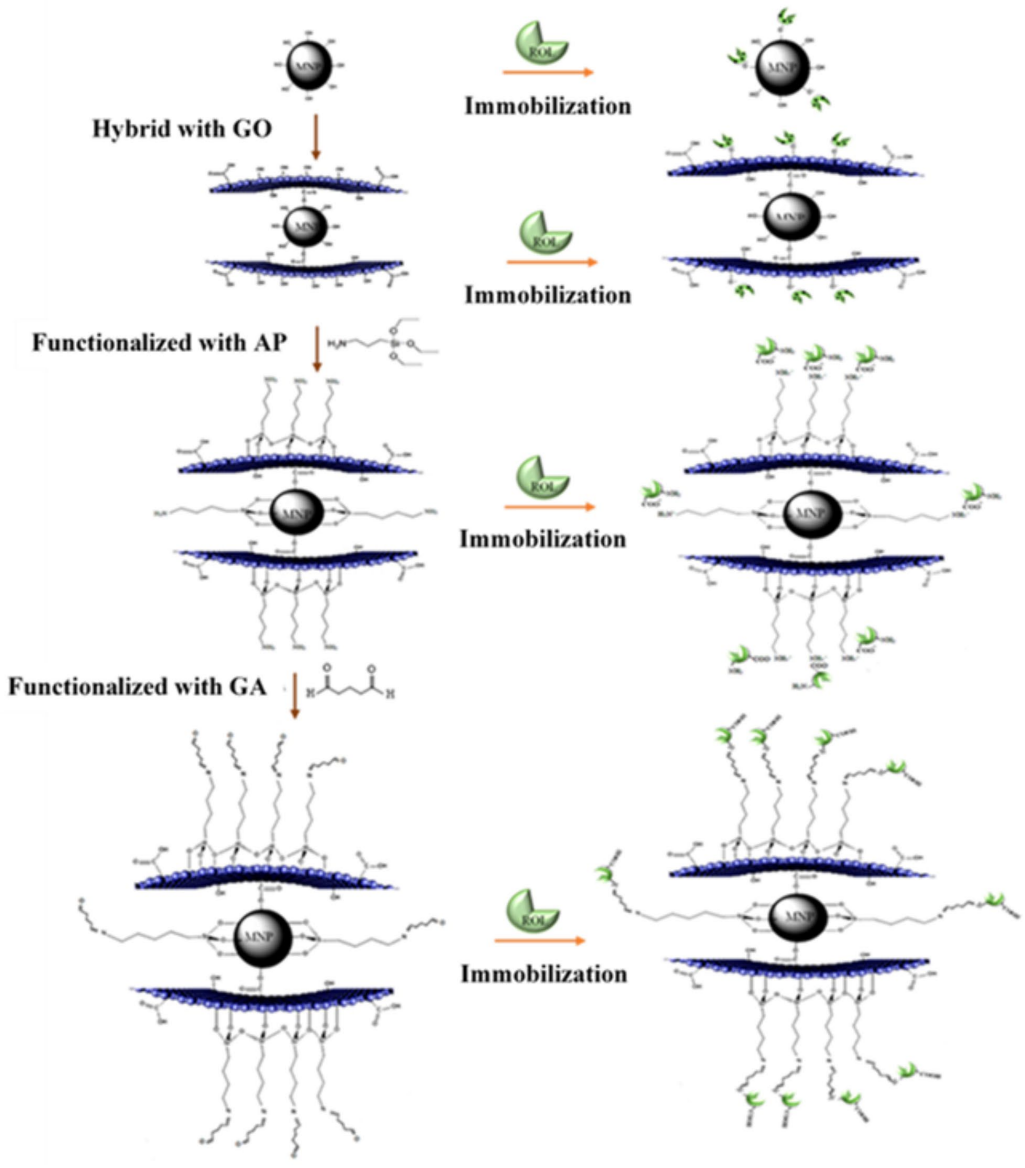
A schematic view of support preparation and enzyme immobilization

In a typical enzyme immobilization experiment, 5 mg of each supports (MNP, MGO, MGO–AP, MGO–AP–GA) were suspended to 1 mL phosphate buffer (100 mM) at pH = 7.5 and then added to ROL solution with different concentrations. The samples were shaken at 4 ℃ for 17 h in a shaking incubator at a stirring speed of 120 rpm. The solids were isolated from the suspension under a magnetic field and then washed five times with the buffer solution. A schematic view of support preparation and enzyme immobilization is presented in Fig. 14. The loaded protein content was measured with Bradford’s method using BSA as a standard. The loading capacity (Loading) was defined as Eq. 1:

1 $$ {\text{Loading capacity}}\; = \;\frac{{{\text{Initial ROL weight }}\left( {\text{mg}} \right)\; - \;{\text{not bounded ROL }}\left( {\text{mg}} \right)}}{\text{Weight of support}}\; \times \; 100. $$

#### Lipolytic activity and kinetic parameters

The spectrophotometric enzyme assay was applied to evaluate the lipolytic activity and kinetic parameters of free and immobilized ROL. For this purpose, the enzymatic hydrolysis of *p*-NPP as the model substrate was utilized. Substrate solution was prepared by mixing 1 volume of *p*-NPP solution (16.5 mM *p*-NPP solution in isopropanol) and 9 volume of buffer solution containing 0.04 mL Triton X-100 and 0.01 g Arabic gum in 10 mL PBS; 100 mM. Then, 8.6 mg of ROL was added to a certain amount of substrate solution to start the reaction and kept it 5 min. The release of *p*-nitrophenol was measured using UV–visible spectrophotometry (Varian Cary 100 Spectrophotometer) at 410 nm. One unit of the enzyme (1 U) was defined as the amount of biocatalyst that released 1 µmol of *p*-nitrophenol per minute under the assay condition. The kinetic parameters (*V*_max_ and *K*_*m*_) of all the nano-biocatalysts for *p*-NPP hydrolysis were obtained from Michaelis–Menten equation and Lineweaver–Burk plots [61]. Lineweaver–Burk plots were created by measuring initial velocity (*V*_0_) at different concentrations from 0.1 to 2 mM of the substrate (*S*) in the assay mixtures under the experimental conditions. The turnover number (*k*_cat_) and the specificity constant (*k*_cat_/*K*_*m*_) for all nano-biocatalysts were also determined and compared. The determination of lipolytic activity and kinetic parameters were carried out in triplicate. Statistical analysis was performed to evaluate the differences between the study groups. Analysis of variance (ANOVA) and the least significant difference (LSD) post hoc tests was used for statistical analysis. *P*-values less than 0.05 were considered significant.

#### Time-course thermostability and storage stability

For evaluation of free and immobilized ROL thermostability, all samples were incubated at 60 ℃ over different periods of time (0, 10, 20, 30, 40, 50 and 60 min). The nano-biocatalysts residual activity was measured after cooling in the ice bath for 30 min. The storage stability of free and immobilized ROL was assayed at 4 ℃ and 25 ℃ and residual activity was measured at the gap of 6 days for 30 days period. All nano-biocatalysts residual activities were investigated based on p-NPP hydrolysis in optimum conditions.

#### Biomass cultivation, lipid extraction, and biodiesel production

*Chlorella vulgaris CCAP (211/19)* was cultivated in a nitrogen-starvation modified BG11. Briefly, a modified BG11 culture was achieved in several 4 L glassy tubular photo-bioreactor under continuous 4 Klux cool-white LED with an air pump for 12 days. Microalgae were grown in a modified BG11 medium during the first 6 days. For the second 6 days, cultivation was continued under nitrate-starvation conditions with the aim of increasing biomass lipid content. Afterward, the microalgae were harvested using centrifugation (40 min, 4000 rpm) and washed twice with deionized water. The total lipid content per algal biomass (> 35% dry weight) was determined via the sulpho-phospho-vanillin (SPV) method [62]. The lipid extraction was performed using liquid–liquid extraction.

Transesterification reaction was carried out to convert the extracted microalgae lipid to fatty acid methyl esters (FAMEs) in the presence of immobilized ROL at 45 ℃ for 24 h. In a typical reaction, 8.6 mg of nano-bio catalyst was added to 0.5 mL of extracted lipid dissolved in n-Hexane (5 mg cm^−3^) and 0.15 mL of methanol was added in three reaction time steps of 0, 8, and 12 h to avoid ROL deactivation. At the end of the reaction, two-phase media appeared. The nano-bio catalyst was collected using an external magnetic field from the lower phase (aqueous phase) which consisted of glycerol and other water-soluble components. Subsequently, the upper phase contained FAMEs diluted in hexane was subjected to GC–MS analysis. Biocatalysts were reused in the reactions with the mentioned condition. For reusing experiments, each nano-bio catalyst was washed with hexane for removing the adhered algae oil before using it in the next step. Reaction conversion was defined as the weight ratio of the produced FAMEs to the initial algae oil.

#### Nano-biocatalyst characterization and product analysis

The interactions and the chemical bonding of nanoparticles and enzymes were inspected by Fourier-transform infrared spectroscopy (FTIR) analysis via Bruker Tensor 27 Fourier-transform infrared. The spectra recorded in the range of 4000–400 cm^−1^ using the KBr matrix. The X-ray field emission scanning electron microscopy (TESCAN MIRA3 XMU FESEM) was applied for MNP, MGO, and ROL/MGO–AP–GA surface morphology. Element distribution was also evaluated from the energy dispersive spectrometer analysis (EDS-Element mapping). The identification of crystalline phases in all catalysts was performed by powder X-ray diffraction using a Philips Analytical X-ray diffractometer (XPert MPD) with monochromatized Cu/KÂ radiation. Vibrating sample magnetometer (VSM, Meghnatis Daghigh Kavir Co., Iran) was applied to measuring the magnetization of support. The BET model of Quantachrome Instruments Autosorb-1 was used for the measurement of specific surface area. The degassing was performed at 200 ℃ for 3 h and the adsorption–desorption isotherms were recorded at 77 and 298 K under nitrogen flow. The chemical compositions of product mixtures were analyzed using a GC–MS system of HP Agilent Technology 6890 Network Gas Chromatogram System with column HP-5MS (Length: 30 m, internal diameter: 0.25 mm, Film thickness: 0.25 µm and Material: Fused silica) and Mass Spectrometer (5973 Network Mass Selective Detector). The carrier gas was He with 99.999% purity and the temperature of the injector was set at 250 ℃. A temperature program was used for the column (Initial temperature: 60 ℃, Initial time: 2 min, Temperature rising rate: 10 ℃ min^−1^ up to 200 ℃ and 5 ℃ min^−1^ to 240 ℃, and Final temperature: 240 ℃ for 7 min). The temperature of the ion source of the mass spectrometer was set at 250 ℃, and the ionization mode was electron impact with an electron energy of 70 eV. The internal standard method was used for the determination of FAMEs and Heptadecanoic acid methyl ester (C18:0) was selected as standard material.

## Data Availability

All data generated or analyzed during this study are included in the manuscript.

## Abbreviations

FAME: Fatty acid methyl esters
ROL: *Rhizopus oryzae* lipase
GO: Graphene oxide
MNP: Magnetic nanoparticles (Fe_3_O_4_)
AP: 3-Aminopropyl triethoxysilane
GA: Glutaraldehyde
MGO: Magnetic graphene oxide
ROL/MNP: ROL immobilized on MNP
ROL/MGO: ROL immobilized on MGO
ROL/MGO–AP: ROL immobilized on MGO–AP
ROL/MGO–AP–GA: ROL immobilized on MGO–AP–GA
p-NPP: p-Nitrophenyl palmitate
BG-11: Blue-Green 11
FESEM: Field emission scanning electron microscopy
EDS: Energy dispersive spectrometer analysis
VSM: Vibrating sample magnetometer
FTIR: Fourier-transform infrared spectroscopy
XRD: X-ray diffraction
AFM: Atomic force microscopy
GC–MS: Gas chromatography–mass spectroscopy
ANOVA: Analysis of variance
LSD: The least significant difference
